# Formulation and Nutritional Evaluation of Instant Vegan Mushroom (*Pleurotus ostreatus*) Soup Powder Enriched with Moringa (*Moringa oleifera*), Mung Bean (*Vigna radiata*), and Pumpkin (*Cucurbita maxima*) †

**DOI:** 10.3390/foods15030445

**Published:** 2026-01-26

**Authors:** Chamodi Pamalka, Melani Raymond, Nadeera Gayan, Iain A. Brownlee, Geethika Savindhi Gammeddegoda Liyanage

**Affiliations:** 1 Department of Food Science and Nutrition, Faculty of Life and Medical Sciences, BMS Campus, Colombo 00600, Sri Lanka; chamodi.p@bms.ac.lk; 2 Department of Biotechnology, Faculty of Life and Medical Sciences, BMS Campus, Colombo 00600, Sri Lanka; melani.raymond@bms.ac.lk; 3 AiGrow (Pvt.) Ltd., Bays 1-5, TRACE Expert City, Colombo 01000, Sri Lanka; 4 Department of Applied Sciences, Faculty of Health and Life Sciences, Northumbria University, Newcastle-upon-Tyne NE1 8ST, UK

**Keywords:** antioxidants, moringa, oyster mushroom, soup powder, vegan

## Abstract

Although plant-based convenience foods have gained significant market share, many are high in fat, salt, and sugar while low in nutrients. The current study aimed to develop a vegan oyster mushroom soup powder enriched with moringa, mung bean, and pumpkin. These ingredients were chosen for their high nutritional value and availability. Four soup formulas, each containing varying amounts of moringa (0%, 1%, 2%, and 3%), were prepared, and a sensory evaluation, proximate analysis, and total aerobic plate count were carried out. The 1% moringa formulation showed the highest consumer acceptance. In this formula, moisture, ash, protein, fat, fiber, carbohydrate, and energy content were reported as 13.6%, 7.6%, 16.3%, 2.2%, 9.8%, 50.5%, and 287 kcal/100 g, respectively. The novel powdered soup product had higher amounts of phenolic compounds, total antioxidants, and iron compared to local, commercially available equivalents. Total aerobic plate counts remained below 10^5^ CFU/g; a common acceptability limit for dried soups, throughout the 4-month storage study under ambient conditions. Overall, the developed soup powder demonstrated superior nutritional quality and could support consumers in meeting their daily nutrient requirements. With further refinement, particularly by optimizing the drying process to better retain heat-sensitive nutrients, this product shows potential as an affordable and nutritious option to address inadequate protein intake and iron deficiency in Sri Lanka.

## 1. Introduction

Consumer habits continue to change and evolve in relation to food choice. Convenience foods limit the time burden of preparation and procurement but are typically high in fat, salt, or sugar and limited in fiber, vitamins, and minerals [1]. Additionally, a higher proportion of global consumers are opting for plant-based diets [2]. Those consuming higher proportions of plant-based foods (particularly vegans) should ensure that they consume adequate amounts of essential nutrients. Key nutrients of focus include vitamin B12, omega-3 fatty acids, calcium, vitamin D, and iodine. Adequate intake of iron and zinc also requires consideration due to the reduced bioavailability of these minerals from plant sources. Moreover, dietary strategies like enhancing iron absorption with vitamin C and consumption of fortified foods/supplements can help mitigate these risks [3]. While appropriately planned vegan, vegetarian, and flexitarian diets can provide the full gambit of essential nutrients, modern consumers of “plant-based” foods tend to be increasingly reliant on pre-prepared convenience foods [4]. Therefore, there is a growing demand for convenient, nutrient-rich, plant-based foods to meet the dietary needs of vegans/others to limit the risk of nutrient deficiencies. Instant soup powders, which can be reconstituted quickly, offer a realistic solution to this issue. These products are easy to transport, store, and preserve at room temperature. However, many commercially available soup powders are non-vegan and/or do not include nutrients targeted to benefit the nutritional needs of vegans [5]. This creates a gap in the market for vegan-friendly soup options. To address this demand, an innovative approach using oyster mushroom (*Pleurotus ostreatus*), mung bean (*Vigna radiata*), pumpkin (*Cucurbita maxima*), and moringa leaf (*Moringa oleifera*) is proposed, due to the positive nutritional profile of the ingredients.

The oyster mushrooms are widely cultivated in Sri Lanka and globally. They are characterized by low energy (39 kcal/100 g fresh weight) and fat content. Like other edible fungi, oyster mushrooms are nutrient dense, providing relatively high levels of protein (1.04–1.10 g/100 g fresh weight), dietary fiber (2.0–5.5 g/100 g fresh weight, including β-glucans), and an array of vitamins such as riboflavin (B2), niacin (B3), and ergosterol, alongside minerals including K, P, and Fe [6,7]. The proportion of essential amino acids (lysine, leucine) in oyster mushrooms is superior to many other plant proteins, underlining its value as a suitable ingredient for vegan product development [8]. Interestingly, the presence of aspartic acid and glutamic acid in oyster mushrooms is similar to monosodium glutamate (MSG), which gives a unique taste to mushrooms [9]. They also include low (mainly unsaturated) fat content. The levels of digestible carbohydrates in oyster mushrooms are limited; mannitol and glucose are very low (less than 1% dry mass), as is glycogen (5–10% dry mass), resulting in low energy density. β-glucans are found in the cell walls of mushrooms and have been linked to potential benefits to blood lipid profiles, glycemic regulation, and the immune system, among other putative health impacts. As such, oyster mushrooms have a positive compositional profile for vegan food product development due to their balanced profile of nutrients and available bioactive compounds [8].

*Moringa oleifera*, commonly known as the drumstick or moringa tree, is a fast-growing, drought-resistant plant native to the Indian subcontinent and now cultivated throughout tropical and subtropical regions [10]. Traditionally, various parts of the tree, including leaves, pods, seeds, and roots, have been used in culinary applications and as a traditional herbal remedy [11]. Recently, moringa has received attention as a functional ingredient with multiple potential applications and health impacts. From a compositional perspective, moringa has appreciable amounts of vitamins A, B (folic acid, pyridoxine, and nicotinic acid), C, D, and E, as well as Ca, Fe, and Zn. Moringa leaves also contain a range of phytochemicals, including sterols, tannins, flavonoids, alkaloids, saponins, and terpenoids, as well as antioxidant compounds such as chlorogenic acid, gallic acid, kaempferol, and glycosides that could contribute to potential impacts on health [10,12].

Mung beans are a staple in Sri Lankan and wider South Asian cuisine, providing appreciable amounts of protein, starch, fatty acids, and certain vitamins (A, B, C, E) and minerals (Ca, Mg, Fe, K). Additionally, this legume is abundant in bioactive compounds like polyphenols, polysaccharides, and peptides, making it popular as an ingredient to improve nutritional profiles of food. Mung bean protein appears to be highly digestible (73%) and also has essential amino acids that make it a rational ingredient for vegan-friendly, protein-rich soup powder formulations in combination with other ingredients [13].

Pumpkin is a vegetable considered a common source of carotenoids. However, in Sri Lanka, more than 20% of the pumpkin production is wasted during the season due to excess production and post-harvest damage [14]. Considering the sweet taste, aroma, characteristic color, and positive nutrient profile of pumpkin, formulating the vegan soup powder with pleasing organoleptic properties would be an effective approach to minimize the post-harvest losses of pumpkins [15]. As such, this research work aimed to develop a nutritionally positive, novel soup powder using exclusively plant-based ingredients: oyster mushroom (*Pleurotus ostreatus*), mung bean (*Vigna radiata*), pumpkin (*Cucurbita maxima*), and moringa leaf (*Moringa oleifera*). Notably, the primary ingredient, mature oyster mushroom, is an agricultural by-product that is typically considered waste in commercial cultivation due to its tough texture and unmarketable appearance. The utilization of this low-cost biomass provides a fundamental basis for the product’s potential affordability.

## 2. Materials and Methods

### 2.1. Sample Collection

Matured oyster mushrooms were obtained from AiGrow (Pvt) Ltd., Colombo, Sri Lanka. Dried mung beans were purchased from the local supermarket (Keells, Colombo, Sri Lanka). Ripe, fresh pumpkins and moringa were purchased from a local market in Wellawatta, Sri Lanka.

### 2.2. Processing of Mushroom (Pleurotus ostreatus) Powder

First, the mushrooms were cleaned and separated from the fruiting bodies of the bunch. Then, they were sliced to a thickness of 4 mm. The sliced mushrooms were blanched in boiling water for 3 min and then dipped in ice-cold water. After blanching, the mushrooms were dried in the dehydrator (Anywin FD-770, Foshan City, China) at 75 °C for 3 h. This selected condition, based on the manufacturer’s guidelines, successfully yielded fully dehydrated, friable material suitable for subsequent grinding. The dried mushrooms were ground in a mixer grinder (Panasonic MX-AC300, Kadoma, Japan) to a fine powder and sieved through a 0.5 mm sieve. To optimize the preservation of thermally labile bioactive compounds, future research should explore alternative, lower-temperature drying methodologies.

### 2.3. Processing of Mung Bean (Vigna radiata) Powder

The mung bean powder was prepared by roasting it at 160 °C for 20 min until it turned greenish brown. Roasted grams were ground into a powder and sieved through a 0.5 mm sieve.

### 2.4. Processing of Pumpkin (Cucurbita maxima) Powder

Pumpkins were washed and peeled. Then the pumpkin flesh was sliced into small slices (4.5 mm thick). These slices were blanched and then dried in the dehydrator (Anywin FD-770, China) at 75 °C for 3 h. The dried pumpkins were ground in a grinder to a fine powder and sieved through a 0.5 mm sieve.

### 2.5. Processing of Moringa (Moringa oleifera) Powder

Moringa leaves were destalked, boiled at 100 °C for 3 min and transferred to ice-cold water. Then, the blanched leaves were dried for 2 h at 65 °C. The dried leaves were subsequently ground into a powder.

### 2.6. Preparation and Formulation of Mushroom-Moringa-Mung Bean Soup Powder

**Figure 1:**
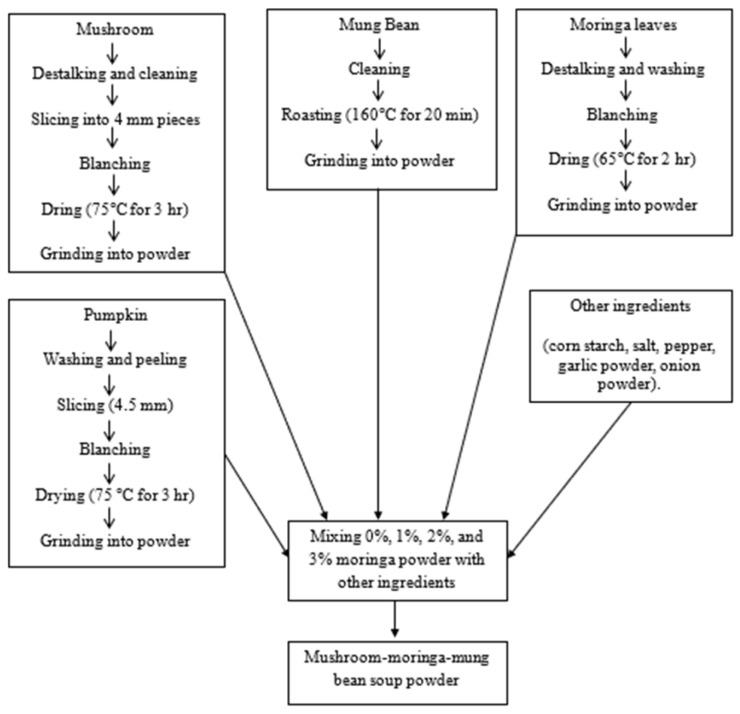
Flowchart for the preparation of mushroom-moringa-mung bean soup powder.

Soup powder was prepared by mixing mushroom powder, mung bean powder, and varying percentages of moringa powder (0%, 1%, 2%, and 3%) with other ingredients (cornstarch, salt, pepper, garlic powder, and onion powder) (Table 1 and Figure 1). The prepared soup powders were then stored in sealed translucent polyethene bags and were stored at room temperature for further analyses.

### 2.7. Cooking Procedure of the Developed Soup Powders

The serving size of the newly developed soup powder was 20 g. The soup mix and water ratio of 1:12.5 (*w*/*v*) was used for the reconstitution of the soup powder. The cooking time (6 min) of the newly developed soup powder was the same as commercial ones.

### 2.8. Sensory Analysis

The sensory attributes, including taste, texture, color, aftertaste, and overall acceptability, were evaluated using a 7-point hedonic scale. The panel consisted of 50 untrained panelists (age range: 18–35 years, including 32 females and 18 males) randomly selected from the staff and students of the BMS campus, Colombo, Sri Lanka. Panelists provided informed consent prior to participation.

For evaluation, the soup formulations were prepared fresh, heated to 65 °C, and presented in 50 mL portions in identical white disposable cups. Samples were labeled with random three-digit codes and served to each panelist in a randomized order. Testing was conducted under the same conditions for each participant, with water at room temperature provided for palate cleansing between samples.

Each attribute was scored based on its intensity using the 7-point hedonic scale (7 = liked very much, 6 = liked moderately, 5 = liked slightly, 4 = neither liked nor disliked, 3 = disliked slightly, 2 = disliked moderately, and 1 = disliked very much). Based on the aggregated sensory scores, the best soup formulation was selected for subsequent proximate analysis, iron and zinc determination, and total aerobic plate count test.

### 2.9. Microbial Analysis

Microbial counts were measured in freshly prepared soup powder with 1% moringa (day 0) and in stored samples at 60, 90, and 120-day intervals. For storage, representative 10 g portions of the soup powder were weighed into 15 individual, high-density polyethylene (HDPE) pouches. The pouches were sealed and stored under ambient laboratory conditions (25 ± 2 °C). At each time point, three pouches were selected randomly and opened aseptically for testing. The Total Aerobic Plate Count technique by the Dilution Pour Plate method, as mentioned in the Bacteriological Analytical Manual [16], was used in the analysis. Plate Count Agar was prepared, mixed with 1 mL of the diluted sample, swirled to ensure even distribution, and allowed to solidify. The dishes were then incubated for 24 h at 37 °C. This temperature was selected as a conservative hygiene indicator, as 37 °C colony counts primarily reflect contamination from human or equipment sources rather than ambient environmental flora. Finally, colonies were counted systematically across the plates. All plating was performed in triplicate, and results are expressed as the mean colony forming units per gram (CFU/g) ± standard deviation (SD).

### 2.10. Proximate Analysis

The proximate composition (moisture, crude protein, crude fat, ash, and crude fiber) of the developed soup powder with 1% moringa was analyzed according to the standard analytical methods [17]. All analyses were performed in triplicate, and results are expressed on a dry weight basis (mean ± SD).

The moisture content was determined by hot air oven drying (AOAC 930.15). Exactly 2.0 g (W_1_) of the sample was placed in a pre-dried and pre-weighed moisture dish (W_0_). The dish was placed in a hot-air oven (Isotherm OFA-54-8, Shanghai, China) and dried at 105 °C until a constant weight was achieved. The dish was cooled in a desiccator and weighed again (W_2_). Moisture content was calculated as follows:

$$Moisture\left(\%\right)=\frac{\left(W_{1}+W_{0}\right)-W_{2}}{W_{1}}\times 100$$

The crude protein content was determined by the Kjeldahl method (Kjeltec 8400, Denmark) (AOAC 2001.11). A 0.5 g sample was digested with 15 mL of concentrated sulfuric acid (98% H_2_SO_4_) and a digestion tablet catalyst at 420 °C for 90 min. The digest was then distilled after the addition of 40% (*w*/*v*) sodium hydroxide (NaOH). The liberated ammonia was trapped in a 4% (*w*/*v*) boric acid solution and titrated with a standardized 0.1 M hydrochloric acid (HCl) solution. A reagent blank was run concurrently. Total nitrogen content was calculated from the titration volume. Crude protein was estimated using a conversion factor of 6.25:

$$Crude\;Protein(\%)=\frac{\left(V_{sample}-V_{blank}\right)\times M_{HCl}\times 0.014\times 100}{Sample\;weight\left(g\right)}\times 6.25$$

where V = titration volume in liters, M = molarity of HCl, and 0.014 = the milliequivalent weight of nitrogen

The crude fat content was determined by solvent extraction using a Soxhlet apparatus (BST/SXW-3, New Delhi, India) (AOAC 2003.05). A 2.0 g sample was weighed into a pre-dried and pre-weighed cellulose thimble. The thimble was placed in the Soxhlet extractor and refluxed with 150 mL of petroleum ether at 50 °C for 6 h. The flask containing the extracted fat was dried in an oven at 105 °C for 30 min, cooled in a desiccator, and weighed. Fat content was calculated as follows:

$$Crude\;Fat\left(\%\right)=\frac{Weight\;of\;fat\;extracted}{Weight\;of\;sample}\times 100$$

The total ash content was determined by dry-ashing samples in a muffle furnace (Sai Scientific, New Delhi, India) (AOAC 942.05). Approximately 3.0 g of the sample was weighed into a pre-ignited and pre-weighed crucible. The crucible with the sample was placed in the muffle furnace at 550 °C for 6 h. The crucible was then cooled in a desiccator and weighed. Ash content was calculated as follows:

$$Total\;Ash\left(\%\right)=\frac{Weight\;of\;ash}{Weight\;of\;sample}\times 100$$

The carbohydrate content was calculated by the difference, and the energy value was determined by multiplying the amount of carbohydrate, protein, and fat by 4.0 kcal/g, 4.0 kcal/g, and 9.0 kcal/g, respectively, and taking the sum of the products [18].

Crude fiber content was determined using the acid and alkali digestion method (AOAC 978.10). Exactly 2.0 g (W_0_) of the defatted soup powder was weighed. For acid digestion, 200 mL of the pre-boiled 1.25% H_2_SO_4_ solution was added to the sample. The mixture was heated to boiling for 30 min with periodic agitation. After digestion, the mixture was filtered, and the residue was washed thoroughly with hot distilled water until the washings were neutral to litmus paper. For alkali digestion, the residue from the crucible was quantitatively transferred back to the original conical flask. 200 mL of the pre-boiled 1.25% NaOH solution was added. A second digestion was performed by boiling the mixture for 30 min. The alkali-digested mixture was filtered. The residue was washed with hot distilled water and collected in a crucible. The crucible containing the residue was dried in an oven at 105 °C overnight until a constant weight was achieved. The crucible was cooled in a desiccator and weighed (P_1_). Then the crucible was transferred to a muffle furnace (Sai Scientific, India) and ignited at 550 °C for 2 h. Finally, the crucible was cooled in a desiccator and re-weighed (P_2_). Crude fiber content was calculated as follows:

$$Crude\;fiber\left(\%\right)=\frac{P_{1}-P_{2}}{W_{0}}\times 100$$

### 2.11. Determination of Iron (Fe) and Zinc (Zn) Trace Elements

The iron (Fe) and zinc (Zn) content in the soup powder with 1% moringa leaf powder was quantified using flame atomic absorption spectroscopy (FAAS) (AOAC 999.11). For digestion, microwave-assisted acid digestion (MARS 6 (240/50), Matthews, NC, USA) procedure was used. A 0.3 g portion of the sample was weighed into a Teflon digestion vessel. Then, 7 mL of concentrated nitric acid (HNO_3_, 65%) and 1 mL of hydrogen peroxide (H_2_O_2_, 30%) were added. After that, the main digestion step was carried out at 190 °C for 20 min. After cooling, the digestate was quantitatively transferred and diluted to a final volume of 50 mL with deionized water. To ensure the method’s accuracy, the NIST 1573a Tomato Leaves reference sample (recovery %−90.3) was processed simultaneously with each digestion. Sample digestion was performed in triplicate (*n* = 3). In FAAS (Thermo Fisher Scientific, Waltham, MA, USA), the instrumental parameters for each element were optimized as follows: wavelengths of 248.3 nm (Fe) and 213.9 nm (Zn), with respective slit widths of 0.2 nm and 1.0 nm. A nitrous oxide-acetylene flame was used for Fe determination, while an air-acetylene flame was used for Zn. Quantification was achieved by calibration with standard solutions prepared in 5% HNO_3_, with concentration ranges of 0.5–5.0 mg/L for Fe and 0.1–2.0 mg/L for Zn.

### 2.12. Estimation of Fatty Acid, Amino Acid, and Vitamin Profiles

The fatty acid, amino acid, and vitamin profiles of the developed product were estimated using compositional data from the Indian Food Composition Table (2017) [19], with nutrient values adjusted for moisture loss during dehydration using standard yield factors. Vitamin retention was accounted for by applying FAO/INFOODS retention factor guidelines [20], including 90% retention for vitamin A (as carotenoids) and thiamine, 100% for vitamins D and E, 80% for vitamin C, 95% for riboflavin and niacin, and 70% for folate. Total nutrient content was calculated by summing the adjusted contributions from all five major ingredients (Oyster mushroom powder, Green Gram powder, Pumpkin powder, Corn flour, and Moringa).

### 2.13. In Vitro Antioxidant Assays

#### 2.13.1. Extraction of Phenolic Compounds and Antioxidants from Soup Powder

The extraction was performed with warm distilled water [21]. Exactly 1.0 g of sample was weighed and mixed with 5 mL of distilled water at 45 °C in a 10 mL centrifuge tube. The tube was shaken vigorously for 5 min and was incubated for 30 min at 4 °C. Then it was centrifuged at 4000 rpm for 10 min (Yingtai Td5, Changsha, China) at 4 °C. The supernatant was carefully collected, and the residual pellet was re-extracted with 2 mL of warm water (45 °C) under the same agitation and centrifugation conditions. The two aqueous extracts from each sample were pooled, filtered, and stored at −18 °C until further analysis. Three extractions were performed per sample.

#### 2.13.2. Determination of Total Phenolic Content (TPC) of Formulated Soup Powders and Locally Purchased Soup Powders

To quantify the TPC of samples, 0.02–0.1 mg/mL of Gallic acid standards were prepared. Each of the diluted soup samples (10X) and standard solutions were added to different test tubes in triplicate along with distilled water as the blank, to which 1.2 mL of 10% Folin-Ciocalteu reagent and 1.5 mL of 7.5% Na_2_CO_3_ were added. The tubes were incubated at room temperature for 60 min. After the incubation, absorbance was measured at 765 nm using a UV-visible spectrophotometer (JENWAY, Felsted, UK). The phenolic concentration was calculated using the standard curve obtained from the Gallic acid standard series. TPC was expressed as equivalents of Gallic acid (mg GAE/100 g) [22].

#### 2.13.3. Determination of Antioxidant Activity of Formulated Soup Powder and Locally Purchased Soup Powders

The 2,2-Diphenyl-1-picrylhydrazyl (DPPH) radical scavenging assay was used to determine the antioxidant activity of the products. A solution of 0.004% DPPH was prepared using methanol. Soup water extracts were dissolved in methanol to obtain a series of concentrations (0.02–0.1 mg/mL). One mL of the soup water extracts at different concentrations and 2 mL of 0.004% DPPH solution were mixed. The mixture was then incubated in the dark for 30 min at room temperature. After incubation, the absorbance was measured at 517 nm using a UV-visible spectrophotometer (JENWAY, UK) against methanol as the blank. The control consisted of DPPH solution mixed with methanol. The percentage of inhibition was calculated using the equation below [23].

$$\mathrm{DPPH}\;\mathrm{Inhibition}\;\mathrm{Percentage}=\left(\frac{Absorbance\;of\;Control-Absorbance\;of\;Sample}{Absorbance\;of\;Control}\right)\times 100$$

The half maximal inhibitory percentages (IC_50_), defined as the concentration of sample required to inhibit 50% of DPPH radicals, were measured using a graph plotted with the inhibition percentage against concentrations of each sample. The IC_50_ was calculated using regression analysis from the resulting inhibition curve.

#### 2.13.4. Determination of Total Antioxidant Capacity (TAC) of Formulated Soup Powder and Locally Purchased Soup Powders

The phosphomolybdenum assay was used to determine the TAC. First, 0.02–0.1 mg/mL of Ascorbic acid standards were prepared. Then, 0.3 mL of soup extracts and each of the standard solutions were added to test tubes in triplicate along with distilled water as the blank, to which 3 mL of the phosphomolybdenum reagent (0.6 M sulphuric acid, 28 mM Sodium Sulphate, and 4 mM ammonium molybdate) was added. The tubes were covered with foil and incubated in a water bath for 90 min at 95 °C. After incubation, the samples were left to cool down, and absorbance was measured at 695 nm using a UV-visible spectrophotometer (JENWAY, UK). The TAC was expressed as equivalents of Ascorbic acid (mg AAE/100 g) [24].

### 2.14. Statistical Analysis

Data obtained from sensory analysis, TPC, DPPH, and TAC were analyzed using One-way ANOVA and Tukey’s multiple comparison analysis test using SPSS software version 27. All data are expressed as the Mean ± Standard Deviation (SD). A *p*-value < 0.05 was considered statistically significant.

## 3. Results and Discussion

### 3.1. Sensory Evaluation of Developed Mushroom-Moringa-Mung Bean Soup Powders

**Figure 2:**
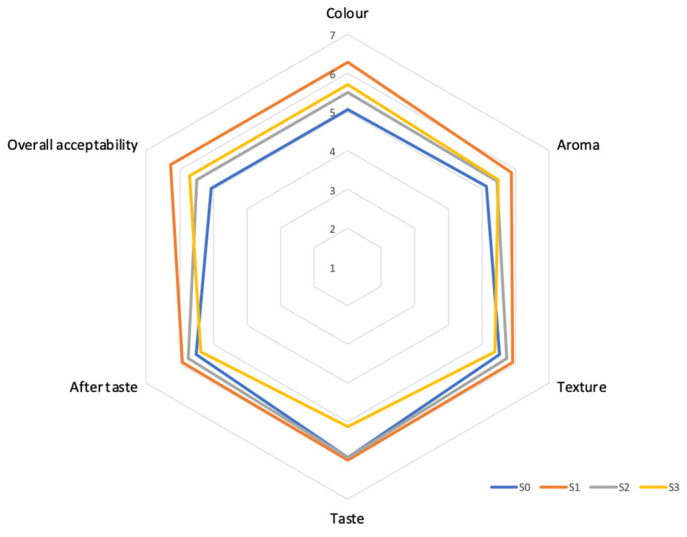
Radar chart of mean scores obtained for the sensory attributes of four soup formulas (S0—0% Moringa, S1—1% Moringa, S2—2% Moringa, and S3—3% Moringa). Each attribute is rated on a scale from 1 to 7 (Scale: 7—Like very much, 1—Dislike very much), with higher scores indicating more favorable sensory qualities.

Figure 2 shows the mean sensory scores obtained for the four developed soup formulations.

Sensory data for all formulations are presented in Table 2. Statistical analysis revealed that S1 scored significantly higher (*p* < 0.05) in color and overall acceptability than the control (S0). For taste, S1 was rated significantly higher than S3 (3% moringa). No significant differences (*p* > 0.05) were found among the samples for aroma, texture, or aftertaste. These results indicate that the addition of 1% moringa leaf powder positively influenced the product’s visual appeal and overall liking compared to the control, without introducing detectable negative flavors or aromas at this concentration.

The higher mean color score for S1 aligns with literature suggesting that low levels of moringa powder enhance visual appeal due to chlorophyll and carotenoid pigments [25]. The optimal overall acceptability of S1, alongside the finding that higher moringa levels (S3) led to a significantly lower taste score, supports the concept of a threshold for beneficial inclusion. As noted by Balogun and team [26], moringa concentrations exceeding 1–2% can introduce bitterness from compounds like tannins and saponins, which may explain the decline in taste scores observed in S3. Therefore, S1 was selected as the optimal formulation for subsequent analysis. While S1 is not statistically different from S2 in overall acceptability, S1 received the highest mean scores for this parameter and for color, representing the most sensorially preferred and ingredient-efficient option that successfully incorporates moringa without compromising palatability.

### 3.2. General Microbial Quality Assessment

Table 3 presents the microbial growth of the 1% moringa-incorporated soup powder mix over selected storage intervals.

As a preliminary assessment of microbial stability, total aerobic plate count was used as a key indicator of general quality change during storage. Data in Table 3 highlight a progressive increase in microbial growth in the soup powder mix over the storage period. The aerobic bacterial count in the 1% moringa soup powder mix increases from 3.2 × 10^3^ CFU/g at 60 days to 1.8 × 10^4^ CFU/g at 120 days, indicating growth over time under ambient storage conditions (25 ± 2 °C). The total aerobic bacterial count remained below 10^5^ CFU/g, which is within commonly accepted limits for dried soup powders in terms of general microbial quality [27]. This preliminary data suggests the product’s potential stability in terms of non-specific microbial load. However, since pathogen-specific tests (e.g., for *Salmonella*, *E. coli*, and *S. aureus*) or tests for molds and yeasts were not conducted, this study does not confirm microbiological safety but rather establishes a baseline for quality trends.

A key limitation of this assessment is the absence of water activity measurement, which is critical for an evaluation of microbial stability in soup powders. Therefore, future studies should include water activity analysis alongside targeted pathogen testing to provide a complete microbiological safety profile. Nevertheless, product-appropriate packaging (aluminum foil laminates, metallized polyethylene terephthalate pouches) would be expected to mitigate moisture uptake and extend general microbial quality [28].

### 3.3. Proximate Compositions of the Developed Soup Powder (On Dry Matter Basis)

For market context, the proximate composition of the developed soup powder was compared against the declared nutritional values from the labels of four commercially available, mushroom-based instant soup powders (Table 4). The commercial products, listed in Table 5, were selected as they represent common local market alternatives. This comparison serves as a practical benchmark rather than a controlled analytical comparison.

#### 3.3.1. Moisture

Moisture content is an important factor in maintaining food quality. A moisture content above 18% encourages the rapid growth of some pathogenic and spoilage-causing microorganisms. Moreover, microorganism growth has been reported to be limited at 8% moisture content in soup powders [29]. However, the moisture content of this newly developed soup powder was 13.6% at the end of the testing period, which was higher than the moringa- and mushroom-based soup powders reported in other studies [30,31]. A study conducted by Farzana and his team [18] noticed a decreased percentage of moisture with an increased percentage of moringa in soup powders. Furthermore, a study done by Sengev’s research team [32] showed that an increase in moringa leaf powder decreased the moisture content of bread. However, the elevated moisture content observed in the developed soup powder in the current study is likely influenced by the time elapsed between product formulation and analysis, as moisture determination was carried out 120 days after preparation rather than immediately after processing. During storage, hygroscopic ingredients, including moringa leaf powder, mushroom powder, and mung bean powder, may absorb atmospheric moisture, thereby increasing the overall moisture content of the product. This highlights the importance of determining the initial moisture content of individual ingredients prior to formulation. In future studies, ingredients with initial moisture contents exceeding 10% should be pre-dried, as they may significantly contribute to moisture accumulation during storage [33].

#### 3.3.2. Macronutrients

The protein content of the developed soup powder was determined analytically as 16.3%, which was higher than the results from other studies where mushroom and moringa were the main ingredients [34,35]. Moreover, this result aligns with findings by Farzana et al. [36] for a soy-mushroom-moringa formulation. A comparison with the declared protein content of commercial mushroom soups reveals a 7.7% to 16.7% range in protein. It is important to note that such a comparison has limitations, as label values are derived from different methodologies and are not analytically verified in this study. However, a qualitative examination of the stated ingredients (Table 5) shows the developed soup (S1) lists oyster mushroom powder as the primary component (49.5%), supplemented by other protein-rich materials like green gram powder and moringa [37,38,39]. In contrast, the commercial soups with lower label protein (CS1, CS2) list refined carbohydrates (corn flour, wheat flour) as primary ingredients, with mushroom included as unspecified pieces or a minor component (10% powder in CS2). The higher protein value declared for CS3 (16.7%), despite a low mushroom content (2.5%), is due to other ingredients in its formulation, such as milk solids and hydrolyzed vegetable protein. Therefore, the high protein content of the developed soup powder (S1) can be directly attributed to its formulation.

The carbohydrate content of the developed soup powder (S1) was analytically determined to be 50.5%, which was lower than the declared values for the commercial benchmarks CS1 (70%), CS2 (57.7%), and CS3 (75%). While direct quantitative comparison is limited by methodological differences, the formulation rationale provides a clear explanation for this observation. As indicated in Table 5, S1 is formulated with oyster mushroom powder (49.5%) as the primary ingredient, followed by other low-carbohydrate, high-fiber components such as green gram powder and moringa [9,10,13]. In contrast, the commercial soups list refined carbohydrates (corn flour, wheat flour, and maltodextrin) as primary constituents. CS1 and CS3 list corn flour and wheat flour, respectively, as the first ingredient, which aligns with their higher reported carbohydrate content. Thus, the lower carbohydrate content of S1 can be attributed to its formulation as a mushroom-forward soup powder rather than a starch-based soup.

The fat content of the developed soup powder was 2.2%, which is lower than the label values for CS2 (15.4%) and CS3 (8.3%). The higher fat content in CS2 and CS3 can be reasonably linked to their ingredient lists, which include added fats and oil-based ingredients such as vegetable fat, hydrogenated palm oil, and cheese powder (in CS2). Conversely, S1 contains no added fats or oils; its minimal fat content derives naturally from its ingredients (oyster mushroom, green gram, and moringa).

#### 3.3.3. Energy

A lower energy value was reported in the newly developed mushroom soup powder compared to other commercial soup powders (CS1, CS2, and CS3). This may be due to the lower fat (2.2%) and carbohydrate (50.5%) content in the final product.

#### 3.3.4. Fiber

The analytically determined crude fiber content of the developed soup powder (S1) was 9.8%. This value can be directly attributed to the formulation’s composition. As detailed in Table 5, S1 is formulated with mature oyster mushroom powder as the primary ingredient (49.5%), a material recognized as a good source of chitin and other polysaccharides contributing to dietary fiber [40]. In contrast, the commercial soups CS2 and CS3 declare 0% dietary fiber on their labels. This suggests these products are not formulated to be significant sources of fiber, as they are based primarily on refined carbohydrates (wheat flour, corn starch, and maltodextrin) and contain only a minor proportion of mushroom (10% powder in CS2, and 2.5% in CS3 as part of a vegetable mix) (Table 5).

From a nutritional perspective, a single serving of S1 (20g) would provide nearly 2.0 g of crude fiber. While this represents a modest contribution toward daily recommendations, it is a meaningful increase compared to conventional, fiber-free soup options like CS2 and CS3. Incorporating such a product into the diet can help address the pervasive “fiber gap” [41], especially given that its sensory acceptability may encourage regular consumption, unlike many other higher-fiber functional foods, which tend to have palatability challenges [42].

### 3.4. Zinc (Zn) and Iron (Fe) Content

Iron deficiency has been a major concern in Sri Lanka recently since the local staple foods tend to contain low amounts of bioavailable Fe [43]. Fe-rich, easy-to-prepare foods, like the current formulation, represent a viable means of supporting improved Fe intake. Interestingly, the newly developed S1 soup powder had 5.18 ± 2.5 mg of Zn and 42.1 ± 14.6 mg of Fe per 100 g of soup powder. One 20 g serving of S1 contains 8.42 mg of Fe and 1.04 mg of Zn. Perera and his team [44] have studied locally available Fe-fortified products’ contribution to the recommended daily allowance (RDA) percentage of Fe for children of 15–16 years based on Sri Lankan guidelines. According to that study, the average estimated daily intake of Fe from such products was 5.6 mg, contributing between 14.36 and 18.67% of the RDA. However, according to the results obtained from the current study, one 20 g serving of the newly developed soup powder provides 8.42 mg of Fe and 1.04 mg of Zn. According to Sri Lankan guidelines, the RDA for adolescents aged 15–17 years is 26 mg/day for iron in males and 32 mg/day in females, and 14.2 mg/day for zinc in males and 11.9 mg/day in females [45]. Based on these values, the present findings indicate a contribution of 26.3–32.4% to the daily iron requirement and 7.3–8.7% to the daily zinc requirement.

However, these values represent total mineral content, not bioavailable mineral content. The iron and zinc in this plant-based formulation are primarily in the non-heme form, whose absorption can be significantly inhibited by dietary components such as phytates present in mung beans [46] and polyphenols present in moringa [47] and the relatively high dietary fiber content of the overall mixture. Dietary fiber, particularly insoluble fiber, can bind to minerals and reduce their bioavailability by decreasing transit time and forming physical barriers [48]. Therefore, while the total iron content is high, the physiologically effective contribution to iron status may be lower than the RDA percentage calculated from total content alone. Future research should include preliminary assessments of mineral bioaccessibility.

### 3.5. Estimated Nutritional Composition

In Table 6, the estimated amino acid, fatty acid, and vitamin contents of the formulated soup powder were reported. According to that, developed soup powder has high protein quality and contains appreciable amounts of multiple B vitamins. While the fatty acid profile is promising for heart health due to a high Total Polyunsaturated Fatty Acids content, there is an opportunity to optimize the lipid profile by improving the omega-6 to omega-3 ratio [49]. The current values are estimated values, and actual values may be lower due to bioavailability and degradation while processing. Further investigations are needed to conduct analytical validation of the nutrient profile through standard biochemical testing.

### 3.6. Evaluation of Antioxidant Properties

Comparisons of Total Phenolic Content (TPC), DPPH radical scavenging activity, and Total Antioxidant Capacity (TAC) in developed soup powder formulations and commercial mushroom soup powders are reported in Table 7.

In the current study, statistical analysis confirmed that all newly developed soup powders containing moringa (S1, S2, S3) possessed significantly higher (*p* < 0.05) TPC values than the commercial samples (CS1, CS2). This enhancement is likely attributable to the phenolic compounds present in the key ingredients of the developed soup powder [12,48,50], which are largely absent in the tested commercial products that use refined starch as a major constituent (Table 5). These phenolic compounds also seem to have retained antioxidant properties, as evidenced by the higher TAC in newly developed soup powders compared to commercial products.

In this study, the IC_50_ values of the soup samples varied, with the sample containing the highest (3%) moringa powder (S3) exhibiting the lowest IC_50_ value of 0.19. This indicates the effectiveness in neutralizing free radicals due to its higher content of antioxidant-rich ingredients like moringa. The TPC, DPPH, and TAC values also highlight that the novel formulation had more positive overall antioxidant attributes than the commercially available formulations. While in vitro potential to inhibit reactive oxygen species does not necessarily translate to benefit to human health [51], these findings provide further evidence of the compositional improvement resulting from moringa addition and also highlight a possible mechanism for improved shelf stability in the final product.

## 4. Conclusions

This study successfully developed and evaluated a novel sensorially acceptable plant-based soup powder designed to address protein and iron deficiencies in Sri Lanka. The formulation innovatively utilizes mature oyster mushrooms; a commercial by-product, as the primary protein source, alongside mung bean, pumpkin, and moringa leaf, demonstrating a practical approach to nutrient-dense food development through agricultural waste valorization. Key findings demonstrate that incorporating 1% moringa leaf powder represents the optimal balance, providing the most sensorially preferred and ingredient-efficient formulation. This optimized product offers a substantial Fe and Zn content, with a single serving (20 g) contributing significantly to the RDA of adolescents. These results validate the product’s feasibility as a locally sourced and nutritionally positive food option.

While the study’s reliance on an untrained consumer panel (*n* = 50), while appropriate for assessing overall acceptability under practical research constraints, limits both the statistical power and broad generalizability of the sensory preference results. To strengthen future development, validation with a larger and more demographically diverse consumer population is recommended, and incorporating a trained descriptive panel would enable more detailed sensory profiling. Together, these steps would support the optimization and broader applicability of this promising nutritional product. Despite these limitations, the current study establishes a strong foundation for a promising nutritional intervention with clear potential for public health impact.

## Figures and Tables

**Table 1:** Formulation of mushroom-moringa-mung bean soup powders.

Ingredients	Formulation of the Soup Powders (g) for 100 g			
	S0	S1	S2	S3
Oyster mushroom powder	50	49.50	49.00	48.50
Green Gram powder	15	14.85	14.70	14.55
Pumpkin powder	6	5.94	5.88	5.82
Onion powder	2	1.98	1.96	1.94
Garlic powder	1	0.99	0.98	0.97
Pepper	1	0.99	0.98	0.97
Salt	5	4.95	4.9	4.85
Sugar	2	1.98	1.96	1.94
Corn flour	18	17.82	17.64	17.46
Moringa	0	1	2	3

S0—0% Moringa powder, S1—1% Moringa powder, S2—2% Moringa powder, and S3—3% Moringa powder.

**Table 2:** Mean sensory scores of developed soup powder formulations.

Treatment	Sensory Attribute					
	Colour	Aroma	Texture	Taste	After Taste	Overall Acceptability
S0	5.06 ^a^ ± 1.9	5.14 ± 1.9	5.52 ± 1.9	5.92 ^ab^ ± 1.5	5.52 ± 1.9	5.06 ^a^ ± 1.9
S1	6.28 ^b^ ± 1.6	5.88 ± 1.7	5.92 ± 1.9	5.98 ^a^ ± 1.5	5.92 ± 1.9	6.28 ^b^ ± 1.6
S2	5.50 ^ab^ ± 1.7	5.44 ± 1.5	5.74 ± 1.8	5.92 ^ab^ ± 1.5	5.74 ± 1.7	5.50 ^ab^ ± 1.7
S3	5.70 ^ab^ ± 1.6	5.48 ± 1.6	5.38 ± 1.9	5.12 ^b^ ± 1.8	5.38 ± 1.9	5.70 ^ab^ ± 1.6

Values are presented as mean ± SD (*n* = 50). Means within the same column, those followed by different superscript letters are significantly different (*p* < 0.05). Sensory attributes were rated on a 7-point hedonic scale (Scale: 7-Like very much, 1-Dislike very much), S0: Control (0% moringa), S1: 1% moringa, S2: 2% moringa, S3: 3% moringa.

**Table 3:** Microbial growth (Total Aerobic Plate Count) in 1% moringa-incorporated soup powder mix during selected storage intervals under ambient conditions.

Test Parameter	Results			
Total aerobic bacteria (CFU/g wet basis)	0 days	60 days	90 days	120 days
	4.6 ± 1.0 × 10^2^	3.2 ± 0.5 × 10^3^	5.4 ± 0.8 × 10^3^	1.8 ± 0.8 × 10^4^

**Table 4:** Proximate analysis of developed soup powder with 1% moringa and four locally available soup powders (on a dry matter basis).

	S1	CS1	CS2	CS3
Moisture (g/100 g)	13.6 ± 1.11	NA	NA	NA
Ash (g/100 g)	7.6 ± 0.19	NA	NA	NA
Protein (g/100 g)	16.3 ± 0.60	8.6 *	7.7 *	16.7 *
Fat (g/100 g)	2.2 ± 0.05	0.8 *	15.4 *	8.3 *
Fiber (g/100 g)	9.8 ± 0.77	NA	0 *	0 *
Carbohydrate (g/100 g)	50.5 ± 1.74	70 *	57.7 *	75 *
Energy (kcal/100 g)	287 ± 23	324.4 *	423.0 *	392 *

S1—newly developed soup powder with 1% moringa leaves powder; CS1, CS2, and CS3—mushroom-based soup powders in local market. Values with * are taken from the product label. NA—not available on the product label.

**Table 5:** Characterization of commercial mushroom soup powder samples used for comparative benchmark.

Sample Code	Mushroom Content	Other Key Stated Ingredients	Origin	Remarks
S1	Oyster mushroom powder 49.5%	Green gram powder, pumpkin powder, onion powder, garlic powder, pepper, salt, sugar, corn flour, moringa	Developed soup powder in this study	Formulated with mushroom as a primary ingredient
CS1	Mushroom pieces, quantity and variety unspecified	Corn flour, skimmed milk powder, sugar, salt, natural identical mushroom flavor, maltodextrin	Local	Mushroom listed after primary carbohydrates, mushroom variety unspecified, and product packaging contains button mushrooms
CS2	Mushroom powder (10%)	Wheat flour, skimmed milk, cornstarch, salt, maltodextrin, cheese powder, vegetable fat, flavor enhancers (621, 635), soy sauce powder	Local	Mushroom listed after primary carbohydrates, mushroom variety unspecified product packaging contains button mushrooms
CS3	Dehydrated vegetables—mushroom (2.5%)	Wheat flour, milk solids, salt, dehydrated vegetables—onion, leeks (0.4%), sugar, hydrolyzed vegetable protein, vegetable fat powder mix, hydrogenated palm oil, natural garlic flavor, flavor enhancers (627, 631), and black pepper 0.3%	Imported	Mushroom listed as a component of dehydrated vegetables, mushroom variety unspecified product packaging contains button mushrooms

S1—newly developed soup powder with 1% moringa leaves powder, CS1, CS2, and CS3—mushroom-based soup powders in local market. Ingredients listed for CS1, CS2, and CS3 are taken from product packaging.

**Table 6:** Estimated amino acid, fatty acid, and vitamin profiles of the formulated soup powder (per 100 g).

Essential Amino Acids (g per 100 g of Soup Powder)	
Histidine	0.38
Isoleucine	0.59
Leucine	1.16
Lysine *	0.97
Methionine *	0.22
Phenylalanine	0.68
Threonine	0.60
Tryptophan	0.18
Valine	0.81
Fatty acid profile (g per 100 g of soup powder)	
Total saturated fatty acids (TSFA)	0.30
Total mono unsaturated fatty acids (TMUFA)	0.06
Total poly unsaturated fatty acids (TPUFA)	1.05
Linoleic (C18:2n6)	0.84
α-Linolenic (C18:3n3)	0.06
Vitamin profile (mg per 100g of soup powder)	
Total Carotenoids	2.70 *
Vitamin D2	0.05 *
Vitamin E	0.43 *
Vitamin C	7.98 *
Vitamin B1 (Thiamine)	0.18 *
Vitamin B2 (Riboflavin)	0.16 *
Vitamin B3 (Niacin)	2.31 *
Folate	0.03 *

Values marked with * indicate that retention factors were applied.

**Table 7:** Comparison of Total Phenolic Content (TPC), DPPH radical scavenging activity, and Total Antioxidant Capacity (TAC) in developed and commercial mushroom soup powders.

Treatment	TPC (mg GAE/100 g)	DPPH Radical Scavenging Activity (IC_50_ Value in mg/mL)	TAC (mg AAE/100g)
S0	3.30 ^abc^ ± 1.10	0.76 ^a^ ± 0.01	2.51 ^a^ ± 0.01
S1	4.38 ^bc^ ± 0.15	0.47 ^b^ ± 0.01	2.64 ^a^ ± 0.06
S2	4.56 ^bc^ ± 0.08	0.22 ^c^ ± 0.01	2.76 ^a^ ± 0.02
S3	5.02 ^c^ ± 0.10	0.19 ^c^ ± 0.00	2.86 ^a^ ± 0.04
CS1	1.99 ^a^ ± 0.13	0.88 ^d^ ± 0.01	1.39 ^b^ ± 0.03
CS2	2.82 ^ab^ ± 0.36	0.98 ^e^ ± 0.00	1.33 ^b^ ± 0.26

S0—0% Moringa powder, S1—1% Moringa powder, S2—2% Moringa powder, and S3—3% Moringa powder, CS1 and CS2—Mushroom-based soup powders in the local market. Different letters in the same column for each soup powder indicate statistical difference (*p* < 0.05). Values are presented as mean ± SD.

## Data Availability

The original contributions presented in the study are included in the article. Further inquiries can be directed to the corresponding authors.

## Abbreviations

The following abbreviations are used in this manuscript:

TPC	Total Phenolic Content
DPPH	2,2-Diphenyl-1-picrylhydrazyl
TAC	Total Antioxidant Capacity
RDA	Recommended Daily Allowance
TSFA	Total Saturated Fatty Acids
TMUFA	Total Mono Unsaturated Fatty Acids
TPUFA	Total Poly Unsaturated Fatty Acids

## References

[1] Zeeni N., Abi Kharma J., Malli D., Khoury-Malhame M., Mattar L. (2024). Exposure to Instagram junk food content negatively impacts mood and cravings in young adults: A randomized controlled trial. Appetite.

[2] De Backer C.J., Hudders L. (2015). Meat morals: Relationship between meat consumption consumer attitudes towards human and animal welfare and moral behavior. Meat Sci..

[3] Koeder C., Perez-Cueto F.J. (2024). Vegan nutrition: A preliminary guide for health professionals. Crit. Rev. Food Sci. Nutr..

[4] Gallagher C.T., Hanley P., Lane K.E. (2022). Pattern analysis of vegan eating reveals healthy and unhealthy patterns within the vegan diet. Public. Health Nutr..

[5] Malhotra A., Lakade A. (2025). Analytical review on nutritional deficiencies in vegan diets: Risks, prevention, and optimal strategies. J. Am. Nutr. Assoc..

[6] Manzi P., Aguzzi A., Pizzoferrato L. (2001). Nutritional value of mushrooms widely consumed in Italy. Food Chem..

[7] Ayimbila F., Keawsompong S. (2023). Nutritional quality and biological application of mushroom protein as a novel protein alternative. Curr. Nutr. Rep..

[8] Mau J.L., Lin H.C., Ma J.T., Song S.F. (2001). Non-volatile taste components of several speciality mushrooms. Food Chem..

[9] Devi P.V., Islam J., Narzary P., Sharma D., Sultana F. (2024). Bioactive compounds, nutraceutical values and its application in food product development of oyster mushroom. J. Future Foods.

[10] Islam Z., Islam S.R., Hossen F., Mahtab-ul-Islam K., Hasan M.R., Karim R. (2021). *Moringa oleifera* is a prominent source of nutrients with potential health benefits. Int. J. Food Sci..

[11] Mirihagalla M.K.P.N., Fernando K.M.C. (2021). Medicinal plants use for home remedies in Sri Lanka: A Review. Int. J. Minor. Fruits Med. Aromat. Plants.

[12] Camilleri E., Blundell R. (2024). A comprehensive review of the phytochemicals, health benefits, pharmacological safety and medicinal prospects of *Moringa oleifera*. Heliyon.

[13] Mekkara nikarthil Sudhakaran S., Bukkan D.S. (2021). A review on nutritional composition, antinutritional components and health benefits of green gram (*Vigna radiata* (L.) *Wilczek*). J. Food Biochem..

[14] Ruwanthika K.O.G.H., Munasinghe M.L.A.M.S., Marapana R.A.U.J. (2023). Nutrient analysis of local pumpkin varieties (*Cucurbita* spp.) grown in dry zone of Sri Lanka and development of a value-added product. Vidyodaya J. Sci..

[15] Jesmin A.M., Ruhul A.M., Chandra M.S. (2016). Effect of pumpkin powder on physico-chemical properties of cake. Int. Res. J. Biol. Sci..

[16] Zhang G., Ferguson M., Blodgett R.J. (2001). Aerobic plate count. Bacteriological Analytical Manual.

[17] (2005). AOAC Official Methods of Analysis of Association of Official Analytical Chemistry International.

[18] Farzana T., Mohajan S. (2015). Effect of incorporation of soy flour to wheat flour on nutritional and sensory quality of biscuits fortified with mushroom. Food Sci. Nutr..

[19] Longvah T., Ananthan R., Bhaskaran K., Venkaiah K. (2017). Indian Food Composition Tables 2017.

[20] Tables on Weight Yield of Food and Retention Factors of Food Constituents for the Calculation of Nutrient Composition of Cooked Foods (Dishes). https://www.fao.org/uploads/media/bognar_bfe-r-02-03.pdf.

[21] Morales F.J., Martin S., Acar O.C., Arribas-Lorenzo G., Gokmen V. (2009). Antioxidant activity of cookies and its relationship with heat-processing contaminants: A risk/benefit approach. Eur. Food Res. Technol..

[22] Yim H.S., Chye F.Y., Tan C.T., Ng Y.C., Ho C.W. (2010). Antioxidant activities and total phenolic content of aqueous extract of *Pleurotus ostreatus* (cultivated oyster mushroom). Malays. J. Nutr..

[23] Gonzalez-Palma I., Escalona-Buendia H.B., Ponce-Alquicira E., Tellez-Tellez M., Gupta V.K., Diaz-Godinez G., Soriano-Santos J. (2016). Evaluation of the antioxidant activity of aqueous and methanol extracts of Pleurotus ostreatus in different growth stages. Front. Microbiol..

[24] Kusuma I.W., Arung E.T., Kuspradini H. (2019). The potential of white-oyster mushroom (*Pleurotus ostreatus*) as antimicrobial and natural antioxidant. Biofarmasi J. Nat. Prod. Biochem..

[25] Kannangara P.T., Ratnayake R.H.M.K., Jayawardana R.J.M.C.N.K., Marage H.M.C.K.H., Sarananda K.H. (2018). Sensory attributes and shelf-life evaluation of an instant soup powder fortified with moringa (*Moringa oleifera Lam.*) leaves. J. Food Agric..

[26] Balogun O.O., Deniran I.A., Owolabi K.P., Olawale O.R., Ogundiran T.S. (2025). Evaluation of macronutrient and selected micronutrient contents of peanut butter fortified with moringa leaf powder. Eur. J. Nutr. Food Saf..

[27] Microbiological Requirements for Food Groups. https://faolex.fao.org/docs/pdf/est37807.pdf.

[28] Chen C., Zhang M., Xu B., Chen J. (2023). Improvement of the quality of solid ingredients of instant soups: A review. Food Rev. Int..

[29] Luh B.S., Woodroof J.G., Bors L. (1975). Commercial Vegetable Processing.

[30] Rekha M.N., Yadav A.R., Dharmesh S., Chauhan A.S., Ramteke R.S. (2010). Evaluation of antioxidant properties of dry soup mix extracts containing dill (*Anethum sowa* L.) leaf. Food Bioprocess. Technol..

[31] Rubilar M., Morales E., Contreras K., Ceballos C., Acevedo F., Villarroel M., Shene C. (2012). Development of a soup powder enriched with microencapsulated linseed oil as a source of omega-3 fatty acids. Eur. J. Lipid Sci. Technol..

[32] Sengev A.I., Abu J.O., Gernah D.I. (2013). Effect of Moringa oleifera leaf powder supplementation on some quality characteristics of wheat bread. Food Nutr. Sci..

[33] El Wakeel M.A. (2007). Ultra Structure and Functional Properties of Some Dry Mixes of Food. Master’s Thesis.

[34] Kumar K. (2015). Studies on development and shelf life evaluation of soup powder prepared by incorporation of white button mushroom (*Agaricus bisporus* L.). South. Asian J. Food Technol. Environ..

[35] Farzana T., Mohajan S., Saha T., Hossain M.N. (2016). Development of a healthy soup powder using phytonutrient enriched mushroom-moringa leaf. DIU J. Allied Health Sci..

[36] Farzana T., Mohajan S., Saha T., Hossain M.N., Haque M.Z. (2017). Formulation and nutritional evaluation of a healthy vegetable soup powder supplemented with soy flour, mushroom, and moringa leaf. Food Sci. Nutr..

[37] Koutrotsios G., Mountzouris K.C., Chatzipavlidis I., Zervakis G.I. (2014). Bioconversion of lignocellulosic residues by *Agrocybe cylindracea* and *Pleurotus ostreatus* mushroom fungi assessment of their effect on the final product and spent substrate properties. Food Chem..

[38] Effiong M.E., Umeokwochi C.P., Afolabi I.S., Chinedu S.N. (2024). Assessing the nutritional quality of Pleurotus ostreatus (*Oyster mushroom*). Front. Nutr..

[39] Sharanagat V.S., Kumar P., Patro S., Ghule P.D., Meena S.N.S., Singh L., Kumar Y., Gundev P., Nagar M., Bhadra R. (2019). Influence of germination on physicochemical, thermo-pasting, and antioxidant properties of moong grain (*Vigna radiata*). J. Food Process. Preserv..

[40] Cheung P.C. (2013). Mini-review on edible mushrooms as source of dietary fiber: Preparation and health benefits. Food Sci. Hum. Wellness.

[41] McKeown N.M., Fahey G.C., Slavin J., Van der Kamp J.W. (2022). Fibre intake for optimal health: How can healthcare professionals support people to reach dietary recommendations?. BMJ.

[42] Elleuch M., Bedigian D., Roiseux O., Besbes S., Blecker C., Attia H. (2011). Dietary fibre and fibre-rich by-products of food processing: Characterisation, technological functionality and commercial applications. Rev. Food Chem..

[43] Chathuranga G., Balasuriya T., Perera R. (2014). Anaemia among female undergraduates residing in the hostels of University of Sri Jayewardenepura, Sri Lanka. Anemia.

[44] Perera D.R.G., Gunawardana D., Jayatissa R., Silva A.B.G. (2020). Estimation of iron content and its contribution in iron-fortified food products consumed by school children in Sri Lanka. J. Food Qual..

[45] Dietary Reference Intakes for Sri Lankans. https://mri.gov.lk/wp-content/uploads/2024/02/Dietary-Referance-Intakes-for-Sri-Lanka.pdf.

[46] Dhole V.J., Reddy K.S. (2015). Genetic variation for phytic acid content in mungbean (*Vigna radiata* L. *Wilczek*). Crop J..

[47] Amagloh F.K., Atuna R.A., McBride R., Carey E.E., Christides T. (2017). Nutrient and total polyphenol contents of dark green leafy vegetables, and estimation of their iron bioaccessibility using the in vitro digestion/Caco-2 cell model. Foods.

[48] Torre M., Rodriguez A.R., Saura-Calixto F. (1991). Effects of dietary fiber and phytic acid on mineral availability. Crit. Rev. Food Sci. Nutr..

[49] Elagizi A., Lavie C.J., Marshall K., DiNicolantonio J.J., O’Keefe J.H., Milani R.V. (2018). Omega-3 polyunsaturated fatty acids and cardiovascular health: A comprehensive review. Prog. Cardiovasc. Dis..

[50] Pathak S., Jain B. (2023). Phytochemical analysis of dried *Moringa oleifera* leaf powder. J. Plant Sci. Res..

[51] Pellegrini N., Vitaglione P., Granato D., Fogliano V. (2020). Twenty-five years of total antioxidant capacity measurement of foods and biological fluids: Merits and limitations. J. Sci. Food Agric..

[52] Raymond M.A., Pamalka K.D.C., Nadeera G.A.G., Brownlee I.A., Liyanage G.S.G. Development of a nutritionally and functionally enhanced instant vegan soup using local crops. Proceedings of the 2nd International/7th Biennial Research Symposium, Industrial Technology Institute.

